# q-Powders: a quick test for screening retronasal olfactory disorders with tasteless powders

**DOI:** 10.1007/s00405-021-06849-8

**Published:** 2021-05-04

**Authors:** Michal Pieniak, Anna Oleszkiewicz, Marie Klockow, Ayaho Yoshino, Antje Haehner, Thomas Hummel

**Affiliations:** 1 Department of Otorhinolaryngology, Smell and Taste Clinic, TU Dresden, Fetscherstrasse 74, 01307 Dresden, Germany; 2 Institute of Psychology, University of Wroclaw, ul. Dawida 1, 50-527, Wroclaw, Poland; 3 Department of Otorhinolaryngology, Nippon Medical School Hospital, Tokyo, Japan

**Keywords:** Olfaction, Olfactory disorders, Retronasal olfaction, q-Powders test

## Abstract

**Purpose:**

To investigate the clinical utility of q-Powders—a retronasal identification screening test.

**Methods:**

A total of 156 subjects (92 females, mean age: 54.5 years ± 17.3 years) completed a 3-item q-Powders retronasal identification test and a 16-items Sniffin’ Sticks orthonasal identification test. We analyzed whether the q-Powders test could differentiate between subjects with normosmia and subjects with an olfactory disorder.

**Results:**

Our data indicated that subjects with an olfactory disorder scored lower in the q-Powders test than subjects with normosmia. The analyses revealed q-Powders test sensitivity of 84% and a test specificity of 64.9% with a score of 2 points taken as a cutoff for olfactory disorders.

**Conclusion:**

The 3-item q-Powders retronasal test may be used for screening purposes in clinical research.

**Level of evidence:**

4

## Introduction

The human olfactory system relates to two different pathways for odor perception. Orthonasal olfaction refers to sensing odors from the environment during sniffing whereas retronasal olfaction is responsible for sensing odors from inside the body. i.e. the oral cavity and the lungs when exhaling, from the stomach when belching, or, probably most importantly, flavor while eating [1]. Many methods have been developed to measure orthonasal olfaction. They vary in their complexity [2–4], length [5, 6] or target group [7, 8]. They are used for various purposes, including fast screening for anosmia [9]. In contrast, methods designed for the measurement of retronasal olfaction are limited. There are few established tools used to assess retronasal functions including the ‘taste powders’ [10], ‘tasteless powders’ [11], ‘candy smell test’ [12, 13] or the choanal release of odors [14] (review in [15]).

The psychophysical tests are based on several trials, rendering these procedures time-consuming which ultimately limits the use of retronasal tests in clinical practice. Thus, the development of a shortened version of a retronasal olfaction test appears to be of importance for patient screening and diagnosis. A fast screening tool for retronasal olfactory dysfunction might be particularly useful to support the distinction between olfactory disorders and gustatory disorders which are often confused by patients who report changes in “taste perception” but actually refer to smell dysfunction resulting in loss of flavor perception [16]. A recent study shows that retronasal identification seems to predict health and sociopsychological functioning of dysosmic patients presenting to an ENT department better than orthonasal olfaction [17]. Retronasal olfaction is involved in flavor perceptions so its dysfunction leads to diminished quality of life largely due to the inability to enjoy the socially-shared experience of eating. Thus, the quick screening of retronasal olfaction may also help to focus on non-sensory consequences of smell loss.

A recent study presented the development of a shortened self-administered version of the ‘candy smell test’ consisting of seven items (7-CST) [18]. The 7-CST enables differentiation between anosmia and normosmia. However, we hypothesized that an even shorter screening tool can be proposed to successfully indicate retronasal smell dysfunctions. We present a study aimed to verify whether a 3-item version of the ‘tasteless powders’ test [11] might be used for retronasal screening purposes.

### Ethical statement

The dataset was acquired in accordance with the Declaration of Helsinki on Biomedical Studies Involving Human Subjects within the frame of a retrospective analysis of data. The study protocol was positively reviewed by the Ethics committee at the University Clinic of the TU Dresden (EK 251112006).

## Materials and methods

### Participants

The study participants were 156 people (92 females) whose age ranged from 11 to 84 years (*M* = 54.5, SD = 17.3 years; 26 participants aged < 36 years, 52 participants aged between 36–55 years, 78 participants aged > 55 years). They were recruited from a patient population at the Smell and Taste Clinic at the Department of Otorhinolaryngology of the TU Dresden. The study sample comprised 6 patients with congenital olfactory loss, 58 patients with idiopathic olfactory loss, 47 patients with post-viral olfactory loss, 21 patients with sinonasal olfactory loss, 17 patients with traumatic olfactory loss and 7 patients were diagnosed with other olfactory problems.

### q-Powders

The short retronasal olfaction test—q-Powders was based on the tasteless aroma powders identification task [11]. q-Powders testing set comprised three odors (cinnamon, banana, garlic; Givaudan Schweiz AG, Dubendorf, Switzerland), each of them was presented with flash cards with 6 descriptors each. The task of the participants was to identify the descriptor that best described the flavor. The odors were selected based on results from previous studies [19] where the identification rates of the 3 selected odors were high (> 95%). To reduce the chance that participants identified an odor correctly due to guessing, each odor was presented with 5 distractor items instead of 3 distractors used in the previous studies [9, 18]. Table 1 presents target items and distractor items used in this study. Before the presentation of an odor the participants were instructed to close their eyes and pinch their noses with their fingers. A small amount of the stimulus (approximately 0.05 g) was delivered to the anterior part of participant’s extended tongue (approximately 1.5 cm from the tip of the tongue). Then participants were asked to pull the tongue back into their mouth, move the stimulus in their mouth, unblock the nostrils and exhale air through the nose. Next, the participants were asked to identify the flavor from a list comprising the name of the target flavor and 5 distractor flavor names. Between the trials, the participants rinsed their mouths with water. The total score ranged from 0 to 3. The test (including instructions) was performed in approximately 5 min, thus in a considerably shorter time than the standard 20-item retronasal odor identification test that may take 20 min to complete.

**Table 1:** Target items and distractor items used in q-Powders test

Target item	Distractor items				
Cinnamon	Hazelnut	Coffee	Coconut	Nutmeg	Cocoa
Banana	Apple	Orange	Cherry	Raspberry	Blueberry
Garlic	Curry	Cloves	Pepper	Paprika	Mustard

As an external validity criterion we employed a 16-item orthonasal identification subtest from the “Sniffin’ Sticks” test battery [3]. In this test, participants are presented with odorant-filled felt-tip pens. During a single trial each pen was opened and presented to both nostrils for approximately 3 s. After the presentation participants were asked to identify the odor using a list of four descriptors presented in writing and visually (picture) and read verbally to the participant prior to odor presentation [20]. The total score in this test was the sum of all correct identifications and ranged from 0 to 16 points. Scores lower than 9 indicate functional anosmia, i.e. the complete inability to use olfactory perception in daily life. The score between 9 and 12 indicates hyposmia and score higher than 12 indicate normosmia [2].

### Statistical analyses

Statistical analyses were performed with jamovi 1.2.27 software (The jamovi project, Sydney, Australia) for Windows™. First, we performed *t* tests for independent samples to investigate sex differences in both olfactory measures. Pearson’s *r* correlation between Sniffin’ Sticks odor identification score and q-Powders score was computed to examine the external validity of the q-Powders. Further, *χ*^2^ tests were computed to compare the number of functional anosmic, hyposmic, and normosmic individuals with the q-Powders test outcomes of 0, 1, 2, or 3 correct identifications. In the next step, an analysis of variance with a covariate (ANCOVA) was performed with orthonasal identification test scores as the dependent variable, q-Powders test outcome (4 levels) as a between-subject factor and participant’s age as a covariate. All post-hoc comparisons were Bonferroni corrected. Finally, we calculated the sensitivity, specificity and Cohen’s kappa (*κ*) of the rest results for different cutoffs.

## Results

There were no significant differences between scores of men and women obtained in Sniffin’ Sticks identification test [*t*(154) = 0.298, *p* = 0.766] and in short retronasal test [*t*(154) = − 0.176, *p* = 0.860]. Therefore, all further analyses were performed for men and women together. Sniffin’ Sticks odor identification score was positively correlated with the q-Powders score (*r* = 0.61, *p* < 0.001).

The study sample comprised patients of a Smell and Taste Clinic who presented with various olfactory disturbances. However, some of them scored above 12 points in the Sniffin’ Sticks identification test. This resulted in the classification of these subjects as normosmic for the purpose of this study, yet their overall Sniffin’ Sticks score and medical history indicated a compromised sense of smell. Based on their scores obtained in the identification test 49.4% of participants were classified as functionally anosmic, 26.9% of the participants were classified as hyposmic, and 23.7% of the participants were classified as normosmic. Participants with normosmia more frequently obtained higher scores in the q-Powders test, whereas participants with functional anosmia more frequently scored lower in the short retronasal test, *χ*^2^(6) = 69.1, *p* < 0.001. Table 2 presents the distribution of participants with functional anosmia, hyposmia, and normosmia against their scores in the q-Powders test.

**Table 2:** Numbers of functional anosmic, hyposmic, and normosmic subjects (according to Sniffin’ Sticks identification test scores) scoring 0, 1, 2, or 3 in q-Powders test

Short retronasal test score	Functional anosmia	Hyposmia	Normosmia	Total
0	25	2	0	27
1	32	8	3	43
2	14	19	10	43
3	6	13	24	43
Total	77	42	37	156

**Fig. 1:**
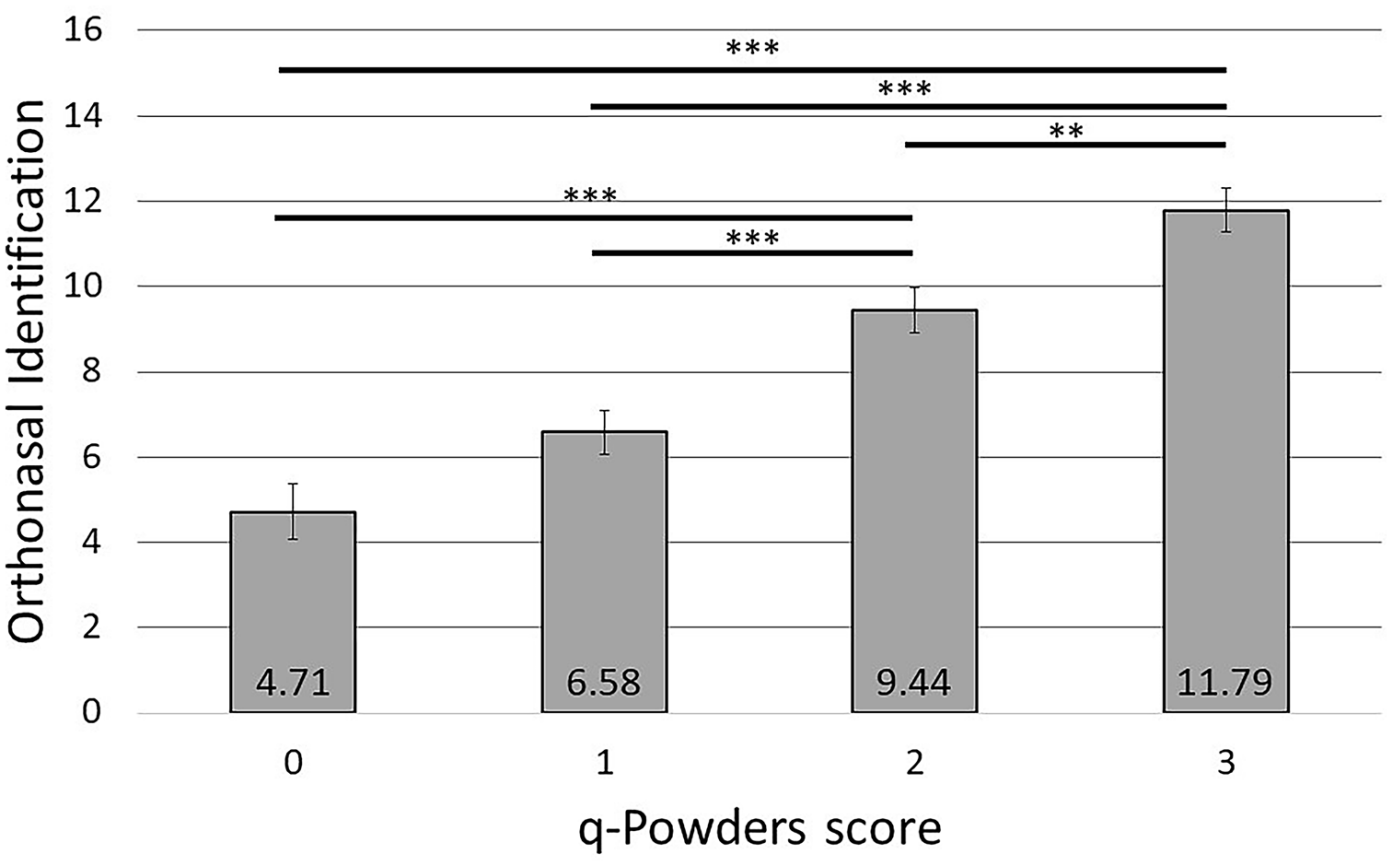
Sniffin’ Sticks identification score in subjects who scored 0, 1, 2, or 3 in q-Powders test. Error bars represent ± SEM; ***—*p* < 0.001, **—*p* < 0.01

The analysis of variance revealed a significant main effect of q-Powders test score, *F*(3, 151) = 30.6, *p* < 0.001, *η*^2^_p_ = 0.38, on Sniffin’ Sticks identification test results. Subjects who scored 0 or 1 points in the short retronasal test had significantly lower results in the Sniffin’ Sticks identification test than subjects who scored 2 or 3 (all *p*s < 0.001). Additionally, subjects who scored 2 points in the short retronasal test had significantly lower results than subjects who scored 3 points (*p* = 0.009). These results are presented in Fig. 1. Participant’s age was not a significant covariate, *F*(1, 151) = 0.05, *p* = 0.82, *η*^2^_p_ < 0.001.

Olfactory dysfunction (anosmia or hyposmia) was distinguished from normosmia with the score of 0, 1, or 2 points in q-Powders test at a sensitivity of 84% and a specificity of 65% (Cohen’s *κ* = 0.46, moderate agreement). The test sensitivity and specificity were similar or even higher for the age groups of < 36 years (sensitivity 83%, specificity 75%, Cohen’s *κ* = 0.56, moderate agreement) and 36–55 years (sensitivity 95%, specificity 67%, Cohen’s *κ* = 0.65, substantial agreement), but were slightly lower for the participants aged > 55 years (sensitivity 78%, specificity 57%, Cohen’s *κ* = 0.29; fair agreement).

We also verified whether q-Powders test is able to differentiate the severity of olfactory dysfunction. Functional anosmia was distinguished from hyposmia and normosmia with a score of 0 points in q-Powders test at a test sensitivity of 32.5% and a specificity of 97.5% (Cohen’s *κ* = 0.30, fair agreement). This agreement was fair when analyzing across age groups of < 36 years, 36–55 years and > 55 years (Cohen’s *κ* = 0.36, 0.21, 0.33, respectively). When we used a score 0 or 1 points to distinguish functional anosmia from hyposmia and normosmia, the test sensitivity was 74% and test specificity was 83.5% (Cohen’s *κ* = 0.58, moderate agreement). In this case, the agreement for the analyzed age groups was moderate (< 36 years: Cohen’s *κ* = 0.69; 36–55 years: Cohen’s *κ* = 0.57; > 55 years: Cohen’s *κ* = 0.54).

Normosmia was identified at the score of 3 points in the short retronasal test at a sensitivity of 64.9% and specificity of 84% (Cohen’s *κ* = 0.46, moderate agreement). The highest agreement was reached for group aged 36–55 years (< 36 years: Cohen’s *κ* = 0.56; 36–55 years: Cohen’s *κ* = 0.65; > 55 years: Cohen’s *κ* = 0.29). Identifying normosmia as a score of 2 or 3 points resulted in a sensitivity of 91.9% and specificity of 56.3% (Cohen’s *κ* = 0.33, fair agreement). The agreement was fair for participants aged between 36 and 55 years (Cohen’s *κ* = 0.39) or > 55 years (Cohen’s *κ* = 0.21). The agreement was moderate for the participants younger than 36 years (Cohen’s *κ* = 0.62).

## Discussion

**Fig. 2:**
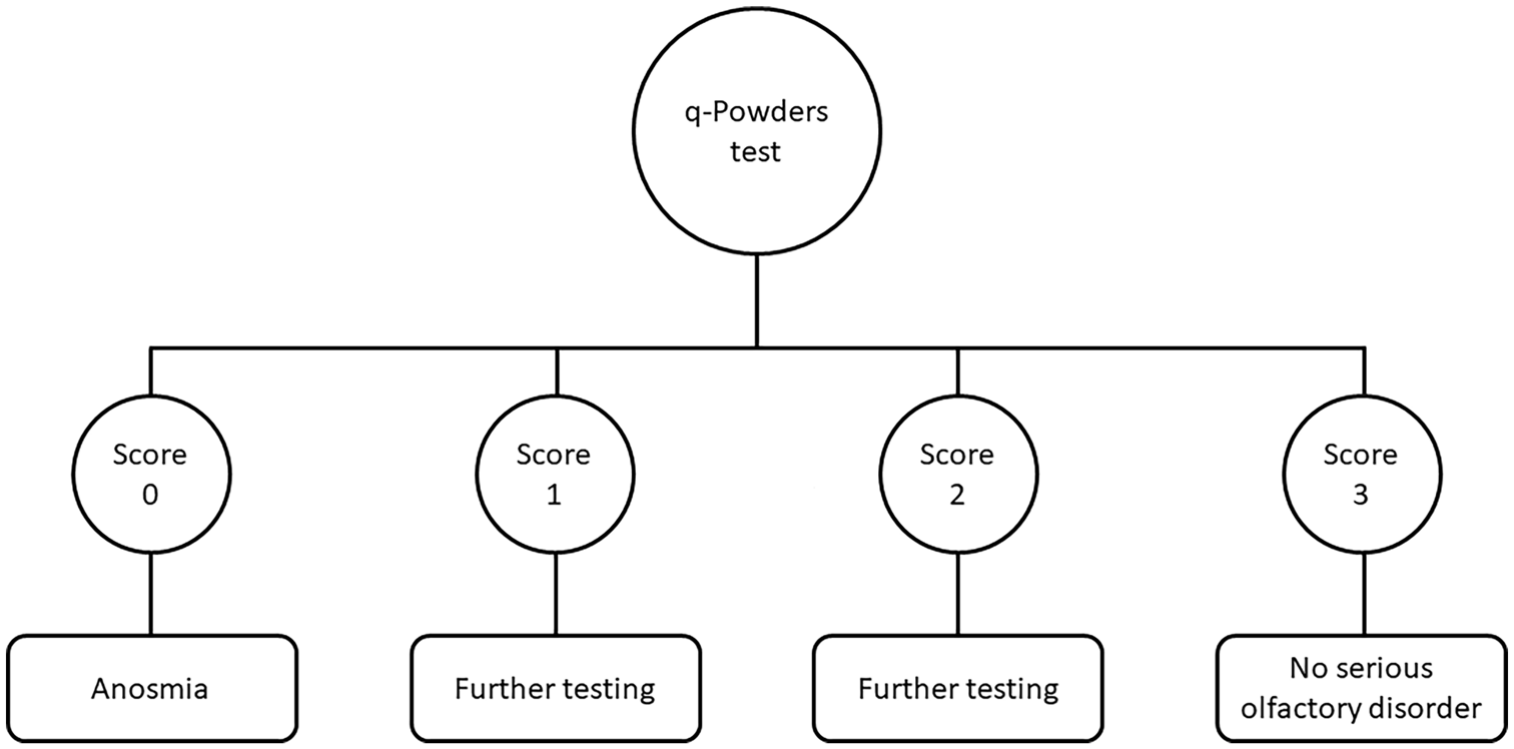
Interpretation of the q-Powders test scores

We tested the q-Powders retronasal test for screening identification dysfunction. The testing set of q-Powders with six descriptors is a valid method that can be recommended as a screening tool for a preliminary diagnosis of retronasal olfactory dysfunction. The data indicated a test sensitivity of 84% and a test specificity of 65% with a cutoff score of 2 points for olfactory dysfunction and score of 3 points suggesting the absence of retronasal olfactory loss (Fig. 2). The proposed screening method should not be used to diagnose retronasal hyposmia, it is meant to differentiate between normal olfactory function and disturbed retronasal olfactory function. However, we recommend that the scores of 1 and 2 points should prompt further in-depth diagnostics, especially in the case of subjects older than 55 years as the lower test specificity (57%) in this group results in a 43% risk of false-positive diagnosis. For all subjects scoring 0 points further testing may be performed to gauge the degree of retronasal olfactory dysfunction.

We also verified that the score of 0 points in the q-Powders test may indicate anosmia. Admittedly, the test sensitivity of 32.5% suggests that people with anosmia often score higher than 0 points, but the very high test specificity of 97.5% indicates that the risk of false-positive diagnosis of dysosmia with a score of 0 points is negligible.

In the q-Powders test, 16% of people with an olfactory disorder can still reach the maximum score of 3 points, being false-negative cases. Approximately 35% of normosmic subjects may score lower than 3 points and will need further olfactory testing to rule out the potential false-positive diagnosis. Thus, the q-Powders test appears to be a valuable tool for screening purposes but it cannot and is not intended to, replace the extended versions of the retronasal olfaction tests [10–12]. The risk of misdiagnosis with the q-Powders is increased in the group of older patients, aged above 55 years. The proportion of false-negative cases rises to 22% in this group due to lower test sensitivity. Hence, older patients with a score of 3 points in q-Powders but who declare subjective distortion of flavor perception should undergo in-depth diagnosis of olfactory functioning. Additionally, based on the clinical work showing discrepancies between the orthonasal and retronasal routes we argue that patients with orthonasal anosmia who score 3 points in q-Powders retronasal test should undergo complete and detailed retronasal testing. Similarly, people with orthonasal normosmia who score 0 or 1 points in q-Powders should be further tested with the complete version of the retronasal test. Overall, the q-Powders might be successfully used by primary healthcare providers or in clinical research which is not focusing directly on retronasal olfaction, but where anosmia is an exclusion criterion.

The new proposed method advances retronasal testing by using tasteless powders. Previous retronasal tests included stimuli activating not only retronasal olfaction but also the gustatory system [11, 17]. Thus, participants had an additional cue for identifying the flavor being guided by the gustatory sensation. q-Powders test resolves this limitation and measures mere retronasal olfaction. Comparing with the extended 20-item version of this test, q-Powders are cost and time efficient, thus likely to be incorporated into medical screening procedures. In our view, the development of chemosensory screening methods is of high importance during COVID-19 pandemic. The characteristic symptom of SARS-CoV2 infection is chemosensory loss [21–24] and considering cumulative growth of cases around the globe and the recurring waves of infections, rapid, cost-efficient methods to diagnose chemosensory disturbances may facilitate the prevention of disease spread and counseling/medical treatment of patients with olfactory loss.

As the retronasal olfactory sensitivity differs among cultures [19], the q-Powders test should be reinvestigated for use in countries other than Germany. Any shortened version of an olfactory identification test should comprise odors which are common in the targeted population. Additionally, the presented study could be supplemented with the test–retest reliability measure of q-Powders.

## Conclusions

Classical retronasal olfactory tests are time-consuming, thus not suitable for screening purposes. We tested whether a short 3-item q-Powders test might be used to identify subjects with anosmia. Our results suggest that in most cases q-Powders screening test correctly differentiates between normosmic individuals and subjects with an olfactory disorder.
